# Analysis of the kinetic mechanism of recombinant human isoprenylcysteine carboxylmethyltransferase (Icmt)

**DOI:** 10.1186/1471-2091-5-19

**Published:** 2004-12-29

**Authors:** Rudi A Baron, Patrick J Casey

**Affiliations:** 1 Department of Pharmacology and Cancer Biology, Duke University Medical Center, Durham, NC 27710, USA; 2 Imperial College London, Division of Biomedical Sciences, Sir Alexander Fleming Building, South Kensington, London SW7 2AZ, UK

## Abstract

**Background:**

Isoprenylcysteine carboxyl methyltransferase (Icmt) is the third of three enzymes that posttranslationally modify proteins that contain C-terminal CaaX motifs. The processing of CaaX proteins through this so-called prenylation pathway via a route initiated by addition of an isoprenoid lipid is required for both membrane targeting and function of the proteins. The involvement of many CaaX proteins such as Ras GTPases in oncogenesis and other aberrant proliferative disorders has led to the targeting of the enzymes involved in their processing for therapeutic development, necessitating a detailed understanding of the mechanisms of the enzymes.

**Results:**

In this study, we have investigated the kinetic mechanism of recombinant human Icmt. In the reaction catalyzed by Icmt, S-adenosyl-L-methionine (AdoMet) provides the methyl group that is transferred to the second substrate, the C-terminal isoprenylated cysteine residue of a CaaX protein, thereby generating a C-terminal prenylcysteine methyl ester on the protein. To facilitate the kinetic analysis of Icmt, we synthesized a new small molecule substrate of the enzyme, biotin-S-farnesyl-L-cysteine (BFC). Initial kinetic analysis of Icmt suggested a sequential mechanism for the enzyme that was further analyzed using a dead end competitive inhibitor, S-farnesylthioacetic acid (FTA). Inhibition by FTA was competitive with respect to BFC and uncompetitive with respect to AdoMet, indicating an ordered mechanism with SAM binding first. To investigate the order of product dissociation, product inhibition studies were undertaken with S-adenosyl-L-homocysteine (AdoHcy) and the N-acetyl-S-farnesyl-L-cysteine methylester (AFCME). This analysis indicated that AdoHcy is a competitive inhibitor with respect to AdoMet, while AFCME shows a noncompetitive inhibition with respect to BFC and a mixed-type inhibition with respect to AdoMet. These studies established that AdoHcy is the final product released, and that BFC and AFCME bind to different forms of the enzyme.

**Conclusions:**

These studies establish that catalysis by human Icmt proceeds through an ordered sequential mechanism and provide a kinetic framework for analysis of specific inhibitors of this key enzyme.

## Background

Posttranslational modification of eukaryotic proteins with lipids is a prevalent mechanism for controlling the subcellular localization and activity of these proteins [1]. Most proteins terminating in a CaaX sequence (C, cysteine; "a", generally an aliphatic residue; X, the carboxy-terminal residue) are subject to modification by isoprenoid lipids via their ability to serve as substrates for protein farnesyltransferase or protein geranylgeranyltransferase type I [2]. Following covalent attachment of the farnesyl or geranylgeranyl isoprenoid to the Cys thiol of the CaaX sequence, the majority of these proteins are further processed by removal of the carboxyl-terminal aaX residues by an endoprotease termed Rce1 and methylation of the newly-exposed carboxyl group of the isoprenylated cysteine residue by an enzyme termed isoprenylcysteine carboxylmethyltransferase (Icmt) [3,4].

While isoprenoid modification of CaaX proteins is the principal determinant of their membrane targeting, the subsequent steps of proteolysis and methylation are clearly important. Studies performed using small molecule prenylcysteines such as N-acetyl-S-farnesyl-L-cysteine (AFC) and N-acetyl-S-geranylgeranyl-L-cysteine (AGGC) [5] showed that carboxyl methylation is a critical determinant of the hydrophobicity of the farnesylated moiety, whereas in the case of geranylgeranylated counterpart the effect is much less. Similar results were obtained in studies with short prenylated peptides [6]. Ras proteins, which are primarily farnesylated, are largely mislocalized in cells in which the Icmt gene has been disrupted and in cells in which the methylation pathway has been perturbed [7,8], suggesting that this processing step is critical for trafficking and/or stable membrane association. In more biological settings, disruption of either the Rce1 or Icmt genes in mice results in embryonic lethality [9,10], and a very recent study has shown that genetic disruption of Icmt in cells dramatically attenuates their ability to be transformed by the K-Ras oncogene [11]. Altogether, a picture has emerged in which C-terminal methylation of prenylated proteins is thought to contribute substantially to their affinity for cell membranes [12-15], to influence rates of protein turnover [11,16], and to facilitate functional interactions with other proteins [17-19].

Although Icmt is a protein methyltransferase, the enzyme can efficiently modify simple prenylcysteines [6,20-22], a property that has the potential to greatly facilitate kinetic analysis of the enzyme. The only kinetic study of an isoprenylcysteine methyltransferase activity to date was performed prior to the molecular identification of Icmt and involved kinetic analysis of a methyltransferase activity in retinal rod outer segment membranes that was capable of modifying the small molecule substrate N-acetyl-L-farnesylcysteine (AFC) [20]. However, the cloning of mammalian Icmt [23], and the development of an expression system to produce recombinant protein [8], now allows an unambiguous analysis of the properties of this important enzyme. Here we report studies of the kinetic mechanism of recombinant human Icmt produced in Sf9 cells. This analysis was also facilitated by the synthesis of a new small molecule Icmt substrate, biotin-S-farnesyl-L-cysteine (BFC), that allowed development of a facile assay for enzyme activity. Through standard Michaelis-Menten analysis as well as product competition and dead-end inhibitor studies, we demonstrate that catalysis by Icmt proceeds through an ordered sequential mechanism in which the substrate AdoMet binds first and its product AdoHCy is released last.

## Results

### Characterization of the small molecule Icmt substrate, BFC

**Figure 1:**
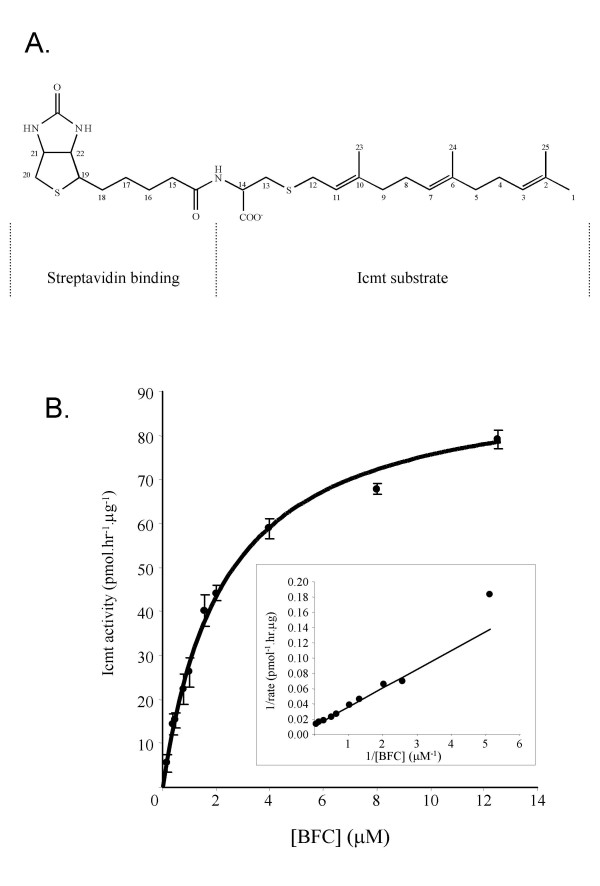
**A new small-molecule substrate of Icmt **A. Structure of biotin-S-farnesyl-L-cysteine (BFC) B. Utilization of BFC as a substrate by recombinant human Icmt. Shown are Michaelis-Menten and Lineweaver-Burk plot (inset) of the formation of BFC-[^3^H]methylester as a function of BFC concentration. The data shown are the mean of duplicate determinations from a single experiment and are representative of two additional experiments.

To overcome the inherent problems associated with use of S-prenylated peptides and proteins as Icmt substrates (expense in production, unwieldy separation techniques, etc), we synthesized a small molecule substrate for the enzyme that contains an appended biotin moiety (Fig. 1A) to facilitate the separation required for product analysis. The coupling of biotin moiety to the free amino group of the S-farnesylcysteine, FC, was readily achieved through the use of commercial biotin N-hydroxysuccinimide ester and the product BFC could be readily purified by precipitation or by reverse-phase chromatography (see *Methods*). BFC was characterized in terms of its ability to serve as a substrate for recombinant human Icmt using a standard *in vitro *methylation assay (Fig. 1B). The apparent K_m _of BFC was 2.1 ± 0.4 μM, which is essentially identical to the K_m _of 2.1 μM determined for farnesylated, Rce1-proteolyzed K-Ras (data not shown). Hence, BFC is a comparable to K-Ras as an Icmt substrate and has the added advantages of being a stable small molecule that can be readily captured on avidin resins.

### Distinguishing ping-pong vs. sequential kinetic mechanisms for human Icmt

**Figure 2:**
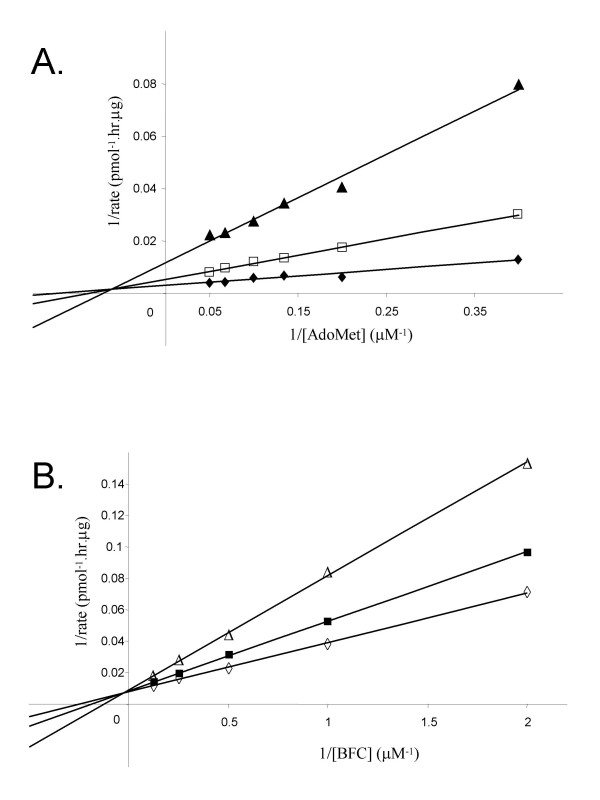
**Distinguishing ping-pong vs. sequential kinetic mechanisms for human Icmt**. A. Lineweaver-Burk plot for the methylation of BFC carried out in the presence of (◆) 2 μM, (□) 4 μM and (▲) 8 μM of BFC and varied [AdoMet]. B. Lineweaver-Burk plot for the methylation of BFC carried out in the presence of (◇) 5 μM, (■) 10 μM, (○) 20 μM of AdoMet and varied [BFC]. For both experiments, the incubation time was 20 min and 0.5 μg of Sf9 membranes containing recombinant human Icmt protein were used for each condition. Data shown are the mean of duplicate determinations from a single experiment, and are representative to two such experiments.

Detailed knowledge of the kinetic mechanism of an enzyme is very important in providing the foundation for analysis of structure-function studies and in inhibitor design studies. For a two substrate enzyme reaction such as that catalyzed by Icmt, there are three basic mechanisms: i) random sequential, ii) ordered sequential, and iii) ping-pong [28,29]. Initial velocity studies can be used to distinguish between a ping-pong mechanism, in which a product is released between the addition of the two substrates, and the sequential mechanisms, in which both substrates must bind before any product is released. Hence, in the first series of studies, the initial velocity of the Icmt reaction at different substrates concentrations was determined. In a ping-pong mechanism for an enzyme with substrates A and B, the plots of 1/v versus 1/[A] at different fixed [B], and vice versa, should yield parallel lines [28]. In a sequential mechanism, either ordered or random, the family of reciprocal plots should intersect above the X axis (if the binding of A increase the interaction with B), below the X-axis (if the binding of A decreases the interaction with B), or on the X-axis (if the binding of A has no effect on the interaction of B) [28]. The data obtained in the experiments where AdoMet concentration was varied at a series of fixed BFC concentrations is shown in Fig 2A, and Fig. 2B shows the data obtained when BFC concentration is varied at fixed AdoMet concentrations. These results show that, in each case, the families of reciprocal plots are not parallel and intersect above the X-axis, ruling out the possibility of a ping-pong mechanism. Moreover, the data for the experiments in which BFC concentration was varied at fixed AdoMet concentrations show an intersection of the reciprocal plots on Y-axis (Fig. 2B), indicating that the reaction behaves as rapid-equilibrium bireactant system [28].

### Distinguishing ordered sequential vs. random sequential mechanisms for human Icmt

**Figure 3:**
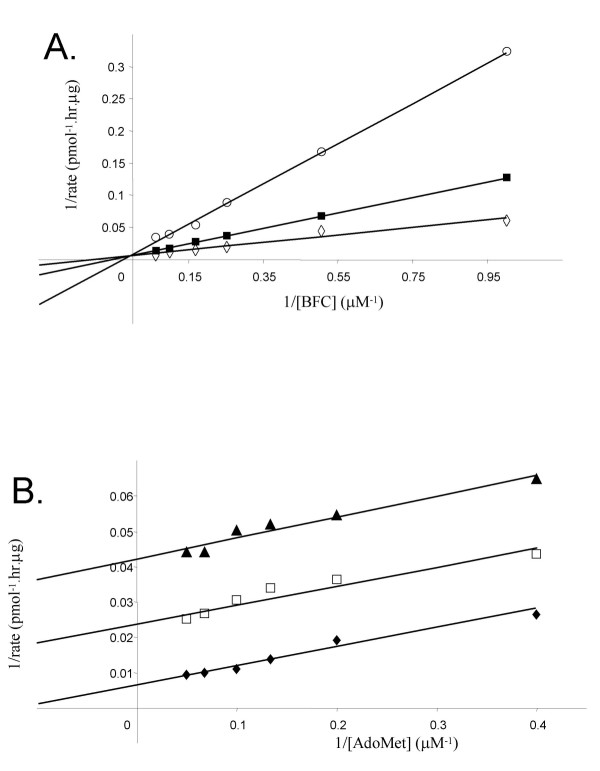
**Distinguishing ordered sequential vs. random sequential mechanisms for human Icmt**. A. Lineweaver-Burk plot for the dead-end inhibition of BFC methylation by FTA at a fixed concentration of AdoMet. Assays were conducted in the presence of fixed [AdoMet] at 5 μM and either (◇) 0 μM, (■) 5 μM or (○) 10 μM of FTA at the indicated concentrations of BFC. B. Lineweaver-Burk plot for the dead-end inhibition of BFC methylation by FTA at a fixed concerntration of BFC. Assays were conducted in the presence of fixed [BFC] at 4 μM and either (◆) 0 μM, (□) 2.5 μM or (▲) 5 μM of FTA at the indicated concentrations of AdoMet. Assays were conducted as described in the legend to Fig. 2. Data shown are the mean of duplicate determinations from a single experiment, and are representative to two such experiments.

While the initial velocity studies described above can distinguish between a ping-pong mechanism and a sequential mechanism, they cannot be used to distinguish an ordered from a random mechanism. These two types of sequential mechanisms can be distinguished, however, through the use of so-called "dead-end" substrates that cannot go on to form products [30]. Hence, we employed such a dead-end inhibitor of Icmt, farnesylthioacetic acid (FTA) [31], in kinetic studies. To distinguish between the two types of inhibition patterns this compound was examined for its inhibitory properties with respect to both the cognate (BFC) and noncognate (AdoMet) substrates under conditions where the fixed substrate was present at nonsaturating concentrations. The results obtained demonstrate that the FTA is a competitive inhibitor with respect to BFC, with a measured K_i _of 1.2 ± 0.2 μM (Fig, 3A, see also Table I). In contrast, the experiments performed with varying AdoMet concentrations show a family of parallel lines (Fig. 3B), which is a characteristic profile for an uncompetitive inhibition [28,29]. If FTA was able to combine with the free enzyme (i.e. the condition where its cognate substrate BFC binds first), the plots in Fig. 3B would have yielded lines that intersect at negative 1/v and 1/[AdoMet] values, i.e. FTA should have shown a mixed-type inhibitor with respect to AdoMet [20]. These results indicate that Icmt reaction occurs through an ordered mechanism in which AdoMet binds first to the enzyme followed by BFC binding to the AdoMet-Icmt binary complex.

**Table 1:** K_i _values and type of inhibition for the interaction of a dead-end inhibitor (FTA) and reaction products (AdoHCy, AFCME) with Icmt. The K_m _values obtained for the two substrates of the reaction, BFC and AdoMet, are also shown for comparaison.

**Substrate\Inhibitor**	**AdoHcy**	**FTA**	**AFCME**
**BFC**	**Competitive**	**Competitive**	**Noncompetitive**
K_m _= 2.1 ± 0.4 μM	3.59 ± 1.03 μM	1.17 ± 0.16 μM	1.91 ± 0.65 μM
**AdoMet**	**Competitive**	**Uncompetitive**	**Mixed-type**
K_m _= 7.8 ± 1.2 μM	3.54 ± 1.12 μM		2.43 ± 0.70 μM

### Distinguishing the order of product dissociation from human Icmt

**Figure 4:**
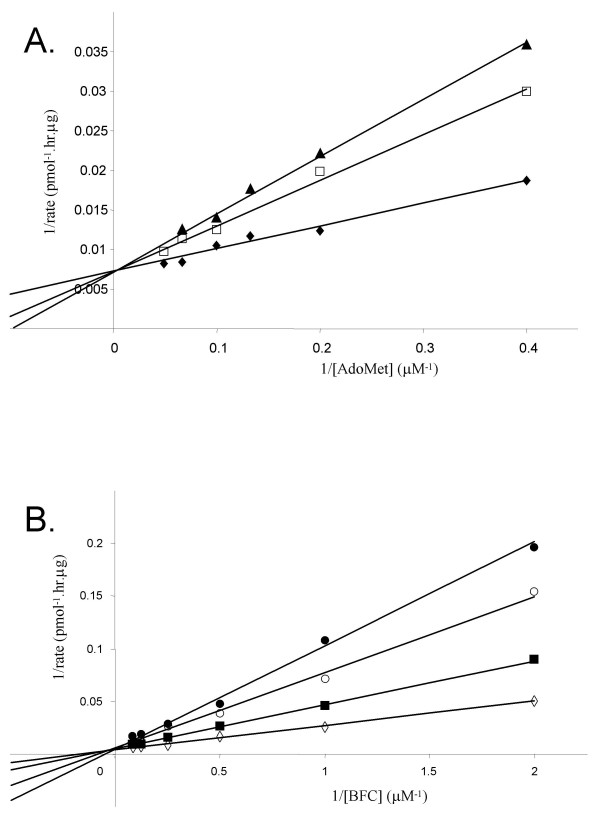
**Distinguishing the order of product dissociation from human Icmt: Inhibition studies with the AdoHcy product**. A. Lineweaver-Burk plot for the product inhibition of BFC methylation by AdoHcy at a fixed concentration of BFC. Assays were conducted in the presence of fixed [BFC] at 4 μM and either (◆) 0 μM, (□) 5 μM or (▲) 10 μM of AdoHcy at the indicated concentrations of AdoMet. Assays were conducted as described in the legend to Fig. 2. Data shown are the mean of duplicate determinations from a single experiment, and are representative to three such experiments. B. Lineweaver-Burk plot for the product inhibition of BFC methylation by AdoHcy at a fixed concentration of AdoMet. Assays were conducted in the presence of fixed [AdoMet] at 5 μM and either (◇) 0 μM, (■) 2.5 μM, (○) 5 μM, or (●) 10 μM μM of AdoHcy at the indicated concentrations of BFC. Assays were conducted as described in the legend to Fig. 2. Data shown are the mean of duplicate determinations from a single experiment, and are representative to more than >4 such experiments.

Two products are formed in the reaction catalyzed by Icmt, a methylated prenylcysteine (*in vivo *on proteins, but *in vitro *also on small molecules or peptides) and AdoHcy. To determine the order of the dissociation of these two products, we performed product inhibition using the prenylcysteine methylester, AFCME, and AdoHcy. Examination of the initial rates of product formation at fixed BFC and varying AdoMet concentrations in presence of three concentrations of AdoHcy (Fig. 4A), revealed that AdoHcy is a competitive inhibitor with respect to AdoMet with an apparent K_i _of 3.5 ± 1.0 μM (Table I). This finding is consistent with both AdoMet and AdoHcy binding to the same form of Icmt, ie. the free enzyme, suggesting that AdoMet binds first in an ordered mechanism and that AdoHcy is the last product released. When the same experiment was performed at varying BFC concentrations, a very similar pattern was observed (Fig. 4B), revealing that AdoHcy is also a competitive inhibitor with respect to BFC with a K_i _of 3.6 ± 1.0 μM. While a classic ordered sequential mechanism in which AdoHcy was released last would result in this type of inhibition being non-competitive or mixed-type, this type of behavior in which a single product binds competitively with both substrates can also be observed in ordered bireactant systems that proceed via a rapid equilibrium process [28].

**Figure 5:**
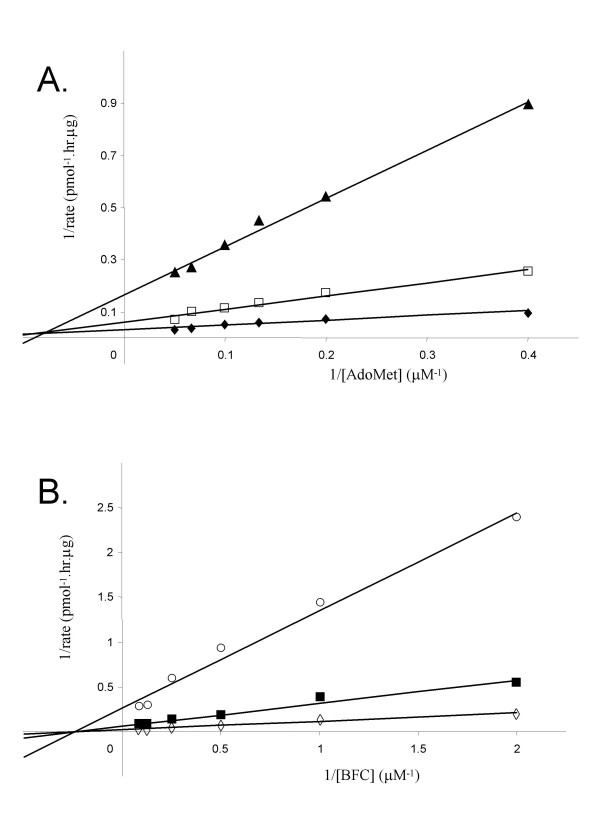
**Distinguishing the order of product dissociation from human Icmt: Inhibition studies with the AFCME product**. A. Lineweaver-Burk plot for the product inhibition of BFC methylation by AFCME at a fixed concentration of BFC. Assays were conducted in the presence of fixed [BFC] at 4 μM and either (◆) 0 μM, (□) 5 μM or (▲) 10 μM of AFCME at the indicated concentrations of AdoMet. Assays were conducted as described in the legend to Fig. 2. Data shown are the mean of duplicate determinations from a single experiment, and are representative to two such experiments. B. Lineweaver-Burk plot for the product inhibition of BFC methylation by AFCME at a fixed concentration of AdoMet. Assays were conducted in the presence of fixed [AdoMet] at 5 μM and either (◇) 0 μM, (■) 5 μM or (○)10 μM of AFCME at the indicated concentrations of BFC. Assays were conducted as described in the legend to Fig. 2. Data shown are the mean of duplicate determinations from a single experiment, and are representative to more two such experiments.

The product inhibition experiments were next repeated with AFCME as the second class of product inhibitor. An ordered sequential mechanism with initial departure of the methylated prenylcysteine product requires that noncompetitive or mixed-type inhibition be observed with respect to both of the substrates of the reaction [29]. The data obtained with L-AFC did indeed demonstrate precisely this [20]. Noncompetitive inhibition by AFCME was observed with respect to the BFC substrate (Fig. 5A) with an apparent K_i _of 1.9 ± 0.6 μM, and mixed-type inhibition was observed with respect to AdoMet as a substrate with an apparent K_i _of 2.4 ± 0.7 μM, respectively (Fig. 5B). These kinetic results are summarized in Table I. In addition, essentially identical results were observed with a second prenylcysteine-type product analog N-acetyl-S-farnesyl-L-cysteine methyl amide (AFCMA) (data not shown). Altogether, the results this kinetic analysis are completely consistent with the reaction catalyzed by Icmt proceeding through an ordered sequential mechanism with the AdoMet substrate binding first and the AdoHcy product being released last.

## Discussion

The studies in this report provide the first detailed kinetic analysis of the molecular entity Icmt, the enzyme responsible for the final step in the maturation of CaaX-type protein in eukaryotic cells. To facility analysis of Icmt activity, we synthesized a new substrate for Icmt, biotin-S-farnesyl-L-cysteine (BFC), that has two properties that improve its utility compared with the only other described small molecule substrate for the enzyme, AFC. First, BFC is utilized by the enzyme with a K_m _(2.1 μM) essentially identical to that of an authentic substrate, farnesylated, Rce1-proteolyzed, K-Ras protein, whereas the apparent Km for AFC is 10-fold higher at 20 μM [20,31]. The second improvement in the BFC substrate is, of course, the appended biotin moiety, which allows facile isolation of product from reaction mixtures using commercially-available avidin resins. Furthermore, this substrate is ideal for use in high-throughput screening for inhibitors of the enzyme since it allows use of scintillation proximity-type assays.

Our first series of kinetic studies was designed to distinguish between the two general kinetic mechanisms that exist for multisubstrate enzymes. The first general mechanism is termed sequential and describes reactions in which all the substrates must bind to the enzyme before the first product is released, whereas reactions in which one or more products are released prior to all substrates being added are termed ping-pong. To distinguish between these reaction types, we performed classic steady state kinetic analyses of Icmt. The results of this analysis revealed that catalysis by Icmt exhibits all the properties of rapid equilibrium bireactant system [28,29] and hence proceeds via a sequential mechanism.

The second series of experiments we undertook was designed to distinguish between a random sequential vs. ordered sequential mechanism; this involved a determination of whether the enzyme could initially form a complex with either substrate or whether must it bind them in a defined order. To verify the assignment of a sequential mechanism for Icmt and to distinguish between an ordered versus a random sequential mechanism, studies with a dead-end inhibitor of Icmt were performed. In a random sequential mechanism, there is no defined order of either substrate binding or product release, whereas in an ordered sequential mechanism the binding of the first substrate is required for the second and the products dissociate in an obligatory order [28,29]. The studies with the dead-end substrate FTA revealed that FTA is a competitive inhibitor with respect to BFC, whereas in a random mechanism FTA would be expected to be a mixed-type inhibitor with respect to AdoMet [28]. Furthermore the double-reciprocal plots from the analysis of FTA as an inhibitor with respect to AdoMet showed the parallel lines indicative of an uncompetitive inhibition, signifying that FTA is unable to bind to the free enzyme. Hence, AdoMet binds first to enzyme and FTA interacts with the AdoMet-Icmt complex since, if BFC combined first with enzyme, the inhibition by FTA should have been competitive with respect to BFC and a mixed-type, noncompetitive or competitive inhibition with respect to AdoMet. Together, these results indicate that the reaction catalyzed by Icmt is proceeds through an ordered sequential mechanism in which the AdoMet substrate binds first.

Following the assignment of a sequential mechanism for Icmt, product inhibition experiments were employed to investigate the order of product dissociation from the enzyme. The finding that AdoHcy is a competitive inhibitor with respect to AdoMet revealed that this compound could interact with free enzyme; ie. it must be the final product released. Also, the finding that the prenylcysteine methylester AFCME exhibited mixed-type inhibition with respect to AdoMet is the result expected if AdoHcy is the last product released. Interestingly, when AdoHcy was examined for its inhibition with respect to BFC a different result was obtained from that of the previous kinetic study of prenylcysteine methyltransferase activity in rod outer segments [20]. With recombinant human Icmt, AdoHcy is a competitive inhibitor with respect to BFC, indicating that the Icmt-AdoHcy complex is inactive and cannot bind to BFC, an observation that further reinforces the conclusion of an ordered sequential mechanism for this enzyme.

The inhibition constants for AFCME determined in our experiments of 1.9 μM and 2.4 μM with respect to BFC and AdoMet, respectively, are substantially different from those in the previous study on the methyltransferase activity in rod outer segments of 41 μM and 73 μM respectively [20]. We believe these results are more likely due to the markedly enriched preparation used in our study rather that to any fundamental difference in the enzyme activities; we used 1000-fold less membrane in our assays and it is likely that the hydrophobic AFCME was substantially "sopped up" by the membrane lipid in the previous study, markedly reducing the level available to interact with the enzyme. This phenomenon, which is related to that known as surface dilution kinetics, comes into play when both the concentration of the membrane lipid and of the ligand employed contribute to the kinetic parameter measured [32].

## Conclusions

In summary, we have demonstrated that the kinetic mechanism of CaaX protein methylation by Icmt is an ordered sequential mechanism in which the AdoMet substrate associates first with the enzyme and the AdoHCy product dissociates last. This work provides a kinetic framework for the analysis of specific inhibitors of this enzyme that will most assuredly be forthcoming given the recent biological validation of Icmt as a target for blocking oncogenic transformation of cells [8,11,33].

## Methods

### Materials

Streptavidin-sepharose beads were purchased from Amersham, *trans*, *trans*-farnesyl bromide, biotin N-hydroxysuccinimide ester and S-(5'-adenosyl)-L-methionine p-toluenesulfonate were purchased from Sigma-Aldrich, L-cysteine was purchased from Novabiochem, [^3^H-*methyl*]-S-adenosyl-L-methionine was purchased from Perkin Elmer Life Sciences, farnesylthioacetic acid (FTA) was purchased from Biomol, S-(5'-adenosyl)-L-homocysteine was purchased from Fluka. The N-acetyl-S-farnesylcysteine methyl ester (AFCME) and N-acetyl-S-farnesylcysteine methyl amide (AFCMA) were synthesized as previously described ([24-26]). Recombinant human Icmt was prepared by infection of Sf9 cells with a recombinant baculovirus containing the entire open reading frame of the human Icmt cDNA as described [8]. The membrane fraction of the infected Sf9 cells, isolated as described for studies with the Rce1 protease [27], was used as the source of Icmt for all studies described.

### Syntheses of biotin-S-farnesyl L-cysteine (BFC)

S-Farnesyl L-cysteine (FC) was prepared by reaction of farnesyl bromide with L-cysteine in methanol/ammoniac solvent as described [26]. The resultant FC product was separated from L-cysteine by extraction with butanol/H_2_O (1:1); the butanol phase was then evaporated under reduce pressure and the FC product washed several times with hexane to remove residual farnesyl bromide.

Two coupling procedures were used to attach the biotin moiety to the amino group of FC; both involved the use of biotin N-hydroxysuccinimide methylester (biotin-NHS) as the biotinylation agent. In the first method, an excess of biotin-NHS (75 μmol) to FC (7.5 μmol) was used. The two compounds were dissolved in 2.3 ml of DMSO and 200 μl of 1M Hepes, pH 12, was added. Following a 2 h incubation at room temperature, the DMSO was evaporated under reduced pressure and the resulting residue extracted with a solution of butanol/H_2_O (1:1). The butanol phase was dried and the residue dissolved in 100 μl of methanol; this solution was then diluted to 1 ml by addition of 0.1% trifluoroacetic acid (TFA). The addition of the TFA resulted in the appearance of a white precipitate of essentially pure BFC as judged by chromatography and NMR analysis.

The second coupling method used an excess of FC (2.5 mmol) compared to biotin-NHS (0.73 mmol); these two compounds were dissolved in 18 ml of DMSO. To this solution was added 2 ml of 1M Hepes, pH 12, and the coupling allowed to proceed for 2 h at room temperature. As with the first method, the DMSO was evaporated under reduced pressure, the resulting residue extracted with a solution of butanol/H_2_O (1:1), and the butanol phase dried and the residue dissolved in methanol. Because of the excess of FC present with the BFC product, specific precipitation of the BFC product was unsuccessful. Instead, the BFC was purified by preparative HPLC on a C_18 _column developed in CH_3_CN/H_2_O (2:3). On this matrix, BFC was well-resolved from FC. The peak fractions were collected and solvent evaporated under reduced pressure; the resulting product was white solid that was >95% pure as judged by analytical reverse-phase HPLC [C_18 _matrix developed in CH_3_CN/H_2_O/TFA (90:10:0.1). Proton NMR spectra of the products obtained by either method were completely consistent with that expected for authentic BFC.

### Icmt assay

The assay developed for Icmt activity involved quantitation of [^3^H]methyl incorporation into the small molecule substrate BFC. For the standard assay, reactions were initiated by addition of Sf9 membranes containing Icmt (0.5 μg protein) to an assay mixture containing BFC (4 μM) and [^3^H]AdoMet (5 μM, 1.3 Ci/mmol) in100 mM Hepes, pH 7.4 and 5 mM MgCl_2 _in a total volume of 45 μl. Reactions were carried out for 20 min at 37°C, whereupon they were terminated by addition of 5 μl of 10% Tween 20. Following termination, streptavidin beads (10 μl of packed beads suspended in 500 μl of 20 mM NaH_2_PO_4_, pH 7.4, contaning 150 mM NaCl) were added, and the mixtures mixed by gentle agitation overnight at 4°C. The beads were harvested by centrifugation in a tabletop microcentrifuge at 10,000 rpm for 5 min and washed 3 times with 500 μl of 20 mM NaH_2_PO_4_, pH 7.4, containing 150 mM NaCl. The beads were then suspended in 100 μl of the same buffer, transferred to scintillation vials, and radioactivity determined. For the kinetic analyses, the concentrations of substrates (AdoMet, BFC) or additional ligands (eg. AdoHcy, AFCME, etc) were varied as detailed in the legends to the appropriate figures.

## Abbreviations used

Icmt, Isoprenylcysteine carboxyl methyltransferase; AdoMet, S-adenosyl-L-methionine; AdoHcy, S-adenosyl-L-homocysteine; AFC, N-acetyl-L-farnesylcysteine; AGGC, N-acetyl-S-geranylgeranyl-L-cysteine; TFA, trifluoroacetic acid; BFC, biotin-S-farnesyl-L-cysteine; FTA, S-farnesylthioacetic acid; AFCME, N-acetyl-S-farnesyl-L-cysteine methylester; AFCMA, N-acetyl-S-farnesyl-L-cysteine methyl amide

## Authors' contributions

RAB carried out all of the studies reported. PJC conceived of the study, and participated in its design and coordination and helped to draft the manuscript. Both authors read and approved the final manuscript.

**Figure 6:**
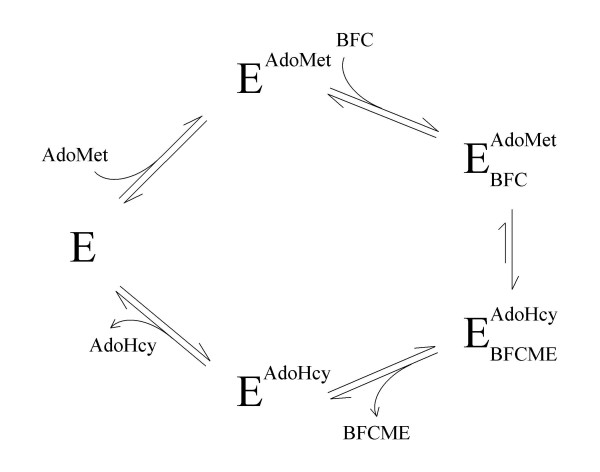
**Proposed kinetic mechanism of human Icmt**. See text for details.
